# Economic impact of pharmacist-led initiatives to minimize medication wastage costs in the hospital setting: an interrupted time series analysis: J. Pharm. Health Care Sci. (Research article)

**DOI:** 10.1186/s40780-026-00612-z

**Published:** 2026-07-28

**Authors:** Tetsuya Ueki, Hidehiro Kamachi, Mariko Oishi, Emiko Sanematsu, Shoko Ogata, Takaharu Ichiki, Kazuhiro Yatera, Fumihiro Tanaka

**Affiliations:** 1 Department of Hospital Pharmacy, Hospital of the University of Occupational and Environmental Health, 1-1, Iseigaoka, Yahatanishi-ku, Kitakyushu, Fukuoka 807-8556 Japan; 2 Department of Respiratory Medicine, School of Medicine, University of Occupational and Environmental Health, 1-1, Iseigaoka, Yahatanishi-ku, Kitakyushu, Fukuoka 807-8555 Japan; 3 Second Department of Surgery, School of Medicine, University of Occupational and Environmental Health, 1-1, Iseigaoka, Yahatanishi-ku, Kitakyushu, Fukuoka 807-8555 Japan

**Keywords:** Medication wastage, Expired drug, High-cost medication, Economic impact, Pharmacist-led initiatives, Interrupted time series analysis

## Abstract

**Background:**

Medication wastage poses significant economic challenges to hospital management worldwide. To address this issue, in October 2020, we implemented comprehensive pharmacist-led initiatives focusing specifically on expired drugs and high-cost medications. This study aimed to evaluate the long-term economic impact of these initiatives using an interrupted time series analysis.

**Methods:**

The comprehensive pharmacist-led initiatives comprised five operational components: proactive deletion of dormant medications from the adoption list, standardization of return and exchange procedures, establishment of a “just-in-time” procurement category, targeted promotion of high-cost near-expiry drugs, and mandatory submission of “High-Cost Medication Waste Reports”. This study compared a 36-month pre-intervention period (October 2017 to September 2020) with a 48-month post-intervention period (October 2020 to September 2024). The primary outcome was the change in total monthly medication wastage costs, evaluated by level change and slope change. Cumulative cost savings over the 48-month post-intervention period were estimated by comparing observed costs with predicted counterfactual values. Secondary outcomes included wastage costs categorized by reason and changes in high-cost medication wastage (50,000 Japanese yen [JPY] or more per case).

**Results:**

No significant level change was observed in total monthly medication wastage costs; however, the slope change was significant (β_3_ = -21,766 JPY/month; 95% confidence interval [CI]: -37,999 to -5,532; *p* = 0.0092). The estimated cumulative cost savings over the 48-month period following the intervention were 38,455,782 JPY. In the analysis by reason, expired drugs showed a significant slope change (β_3_ = -23,820 JPY/month; 95% CI, -34,530 to -13,111; *p* < 0.0001). For high-cost medications, we found significant reductions in wastage costs (β_3_ = -20,001 JPY/month; 95% CI, -35,864 to -4,137; *p* = 0.0141) and the frequency of wastage (incidence rate ratio = 0.977; 95% CI, 0.955 to 0.998; *p* = 0.0355).

**Conclusions:**

Pharmacist-led initiatives focusing on expired and high-cost medications are economically effective, leading to a substantial reduction in hospital medication wastage costs. Pharmacists can enhance hospital financial sustainability by effectively mitigating avoidable medication-related economic losses.

**Supplementary Information:**

The online version contains supplementary material available at https://doi.org/10.1186/s40780-026-00612-z.

## Background

Medication wastage is waste generated in healthcare activities, including drugs that are expired, unused, or no longer needed [1]. The issue of medication wastage is a major global concern that has safety, environmental, and economic aspects [2–6]. In terms of economic losses, a national survey conducted in the United Kingdom estimated prescription drug waste at 300 million pounds sterling each year [2]. Numerous studies in other countries have similarly highlighted the severity of these economic losses [7–11], and the costs have been escalating in recent years with rising drug prices [12].

The causes of medication wastage are manifold, including repeat/habitual dispensing, stockpiling/over-ordering, patient recovery/change of medication, patient death, prescription duration, incorrect disposal, and non-adherence [2]. A systematic review by West et al. identified medication changes, patient death, resolution of conditions, and expired drugs as the most common causes [13]. However, much of the existing evidence relies on self-reports from patients or caregivers and focuses primarily on medication use in the home setting [13, 14]. Evidence regarding hospital-wide medication wastage where drugs are managed by healthcare professionals remains limited, and existing studies are often restricted to quantitative cross-sectional or qualitative designs [15–19].

Although several studies have reported positive results from interventions targeting medication wastage in hospitals, these studies focused only on intravenous drugs [20]; specific medication categories, such as anticancer drugs [21, 22], anesthetics [23], or narcotics [24]; or specific areas such as reducing dispensing errors [25]. There is a lack of research evaluating the impact of interventions on overall medication wastage across an entire hospital setting.

In a previous study, we reported that expired drugs and high-cost medications were the main causes of medication wastage costs in our hospital [15]. Specific examples included expired drugs among infrequently used high-cost medications, unused portions of opened oral anticancer drugs, and wastage of prepared injectable anticancer drugs due to the change or discontinuation of medication after preparation. As the rising cost of medication wastage represents a critical economic challenge for hospital management, we implemented a series of pharmacist-led initiatives to minimize these costs. The aim of this study was to evaluate the economic impact of these comprehensive interventions using an interrupted time series analysis (ITSA), which allows for a robust assessment of changes in both immediate levels and long-term trends while accounting for pre-existing patterns [26–28].

## Methods

### Study design and setting

This study was a single-center ITSA conducted over a 7-year period from October 2017 to September 2024. The study was performed at the hospital of the University of Occupational and Environmental Health, Japan (Kitakyushu, Japan), an advanced treatment hospital with 678 beds. We evaluated the economic impact of pharmacist-led initiatives by comparing the pre-intervention period (36 months from October 2017 to September 2020) with the post-intervention period (48 months from October 2020 to September 2024).

Throughout the study period, the hospital maintained conventional measures to reduce medication wastage. These baseline practices included a “one-in, one-out” policy for formulary adoption, optimizing inventory management to maintain appropriate stock levels, assessing the feasibility of returning or exchanging unused drugs with wholesalers, providing routine information to healthcare professionals to promote the use of near-expiry medications, and basic data collection on medication wastage.

### Enhanced interventions

To further address the challenges of medication wastage and its associated costs, we transitioned to a more proactive approach in October 2020 following approval by the Hospital Management Committee. The enhanced measures implemented since then comprised the following five components, with their specific backgrounds, objectives, target medications, and expected effects summarized in Table 1:

1. Proactive deletion of dormant medications from the adoption list: Medications with no clinical movement for more than one year or those that had expired were targeted for deletion. These candidate medications were selected by pharmacists, and the decision to delete them was approved by the Pharmaceutical Affairs Committee.
2. Standardization of return and exchange procedures: The hospital pharmacy department standardized and strictly enforced the process for returning or exchanging unopened and unused drugs within six months of purchase from wholesalers, with exceptions made for drugs expected to be used within a few days and opened or temperature-controlled products that were ineligible for return under wholesaler policies.
3. Establishment of a “just-in-time” procurement category: A new adoption category was established for infrequently used and non-urgent medications. These drugs were no longer kept in stock at the hospital and were ordered only upon notification of planned use or after a prescription was issued. The selection of drugs for this “just-in-time” category was proposed by pharmacists and approved by the Pharmaceutical Affairs Committee.
4. Targeted promotion of high-cost, near-expiry drugs: Pharmacists proactively provided each clinical department with information on stockpiled medications with an expiry date of less than six months and a potential wastage cost of 50,000 Japanese yen (JPY) or more per case, and personalized this information based on each clinical department’s prescription patterns and clinical characteristics.
5. Mandatory submission of “High-Cost Medication Waste Reports”: In the case of medication wastage cost of 50,000 JPY or more per case, healthcare professionals in the department where the wastage occurred were required to submit a report including the cause and remedial measures. These reports were submitted to the hospital pharmacy department and reported by pharmacists to the Pharmaceutical Affairs Committee for review and confirmation.

**Table 1:** Background, objectives, target medications, and expected effects of the five components of the enhanced interventions

Initiative		Background	Objectives	Target medications	Expected effects
(1)	Proactive deletion of dormant medications from the adoption list	Increase in adopted medications (e.g., drugs approved for specific patients)	Reduction of dead stock	All adopted medications	Reduction in wastage costs due to expired drugs
(2)	Standardization of return and exchange procedures	Increase in total medication purchase amount	Reduction of dead stock	All adopted medications	Reduction in wastage costs due to expired drugs
(3)	Establishment of a “just-in-time” procurement category	Increase in orphan drugs, advancement of personalized medicine, and rising medication costs	Reduction of dead stock	Infrequently used, high-cost medications	Reduction in wastage costs due to expired drugs
(4)	Targeted promotion of high-cost, near-expiry drugs	Increase in orphan drugs, advancement of personalized medicine, and rising medication costs	Reduction of dead stock	High-cost medications (especially opened oral anticancer drugs)	Reduction in wastage costs due to expired drugs
(5)	Mandatory submission of “High-Cost Medication Waste Reports”	Rising medication costs	Prevention of recurrence of high-cost medication wastage	High-cost medications	Reduction in wastage costs due to expired drugs, changes or discontinuations after preparation, preparation errors or accidental damage, and other reasons Raising awareness of high-cost medications among healthcare professionals

### Data collection

Medication wastage data were obtained from an in-hospital database maintained as part of routine clinical practice. These data were compiled from “Medication Waste Reports” submitted by each department. The total cost of medication wastage was aggregated on a monthly basis and calculated using the actual purchase prices at our hospital during the study period. Additionally, the cost and number of high-cost medication wastage cases, defined as those with a cost of 50,000 JPY or more per case, were also counted monthly. The high-cost medications involved in wastage were categorized based on the third level (pharmacological subgroup) of the Anatomical Therapeutic Chemical (ATC) classification system [29]. According to the in-hospital database registration procedure, wastage instances were recorded per individual medication type; thus, a single report containing multiple items was disaggregated into separate entries. For example, if a report documented the wastage of a mixed injection comprising three drug vials and one infusion bottle, these were recorded as two cases. Furthermore, the reasons for medication wastage were classified into the following four groups: (1) expired drugs; (2) change or discontinuation of medication after preparation; (3) preparation errors or accidental damage (such as breakage or contamination due to mishandling); and (4) other.

### Outcome measures

The primary outcome was the change in total medication wastage costs, evaluated through level change (immediate impact after intervention) and slope (change in the long-term trend). Additionally, the cumulative cost reduction over the 48-month post-intervention period was assessed by comparing observed costs with predicted counterfactual values.

The secondary outcomes included changes in medication wastage costs categorized by reason, as well as changes in both the cost and the number of high-cost medication wastage cases. For all cost-based outcomes, level and slope changes were assessed. For the number of high-cost wastage cases, the impact was evaluated using incidence rate ratios (IRRs) derived from the estimated level and slope changes.

### Statistical analysis

We conducted an ITSA using monthly data from October 2017 to September 2024. For the cost-based outcomes, the initial linear regression model included the following variables:

$$\eqalign{ {Y_t}{\rm{ = }} & {\beta _0}{\rm{ + }}{\beta _1} \cdot Tim{e_t}{\rm{ + }}{\beta _2} \cdot Interventio{n_t} \cr & {\rm{ + }}{\beta _3} \cdot Post{\rm{\_}}Slop{e_t}{\rm{ + }}{\beta _4} \cdot Purchase{\rm{\_}}Amoun{t_t} \cr & {\rm{ + }}{\beta _5} \cdot Inpatient{\rm{\_}}Day{s_t}{\rm{ + }}\mathop \sum \limits_{j{\rm{ = }}2}^{12} {\gamma _j} \cdot Mont{h_{jt}}{\rm{ + }}{\varepsilon _t} \cr}$$

The variables in the model are defined as follows:

- $\:{Y}_{t}$ is the monthly total cost of medication wastage at month *t*.
- $\:{Time}_{t}$ is a continuous variable representing the number of months from the start of the study period.
- $\:{Intervention}_{t}$ is a dummy variable indicating the pre-intervention period (coded as 0) and the post-intervention period (coded as 1).
- $\:{Post\mathrm{\_}Slope}_{t}$ is an interaction term between $\:{Time}_{t}$ and $\:{Intervention}_{t}$, representing the time elapsed since the start of the intervention.
- $\:{Purchase\mathrm{\_}Amount}_{t}$ is a covariate for the monthly medication purchase amount to account for fluctuations in hospital activity and drug price revisions.
- $\:{Inpatient\mathrm{\_}Days}_{t}$ is a covariate for the monthly number of inpatient days to account for fluctuations in patient volume and bed occupancy.
- $\:{Month}_{\gamma\:j}$ represents the monthly dummy variables for seasonality (reference: January).
- β_0_ represents the baseline level at the beginning of the study.
- β_1_ estimates the pre-intervention trend (slope).
- β_2_ estimates the immediate change in level following the intervention.
- β_3_ estimates the change in the trend (slope) after the intervention compared with the pre-intervention trend.
- β_4_ estimates the effect of the monthly medication purchase amount on wastage costs.
- β_5_ estimates the effect of the monthly number of inpatient days on wastage costs.
- $\:{\epsilon\:}_{t}$ is the error term.

To optimize the fit of the ITSA model, we performed a stepwise selection process for the primary outcome based on the Akaike Information Criterion (AIC). The initial model including all potential covariates (seasonality, purchase amount, and inpatient days) yielded an AIC of 2191.90. Removing the seasonal dummy variables (*Month*) reduced the AIC to 2185.56. Further removal of the purchase amount (*Purchase_Amount*) decreased the AIC to 2183.62, and the subsequent exclusion of inpatient days (*Inpatient_Days*) resulted in the lowest AIC of 2181.72. Therefore, to prevent overfitting, minimize the risk of multicollinearity, and maintain model stability, seasonality, purchase amount, and inpatient days were excluded from the final model, which was defined as:

$$\eqalign{ {Y_t}{\rm{ = }} & {\beta _0}{\rm{ + }}{\beta _1} \cdot Tim{e_t}{\rm{ + }}{\beta _2} \cdot Interventio{n_t} \cr & {\rm{ + }}{\beta _3} \cdot Post{\rm{\_}}Slop{e_t}{\rm{ + }}{\varepsilon _t} \cr}$$

The results of the models including the monthly purchase amount, either alone or in combination with the monthly inpatient days, along with their variance inflation factors (VIF), are presented as a sensitivity analysis in Supplementary Table 1.

The Durbin–Watson test was used to evaluate the autocorrelation of residuals in the final model, yielding a statistic of 2.427 (*p* = 0.098). No significant autocorrelation was observed, so the model parameters were estimated using ordinary least squares regression. This final model configuration was then consistently applied to all other cost-based secondary outcomes (wastage costs by reason and high-cost wastage costs) to ensure methodological uniformity. The cumulative cost reduction was calculated as the sum of the differences between the predicted counterfactual values and the observed values over the 48-month period.

Regarding the number of high-cost medication wastage cases, we performed an ITSA using Poisson regression with a log-link function to account for the count nature of the data. Following the model selection results for costs, the same variables were used for the Poisson model to maintain consistency across the analyses:

$$\eqalign{ ln\left( {E\left[ {{Y_t}} \right]} \right){\rm{ = }} & {\beta _0}{\rm{ + }}{\beta _1} \cdot Tim{e_t}{\rm{ + }}{\beta _2} \cdot Interventio{n_t} \cr & {\rm{ + }}{\beta _3} \cdot Post{\rm{\_}}Slop{e_t} \cr}$$

Where:

- $\:ln\left(E\left[{Y}_{t}\right]\right)$ is the expected monthly count of high-cost medication wastage.
- The parameters β_1_, β_2_, and β_3_ represent the change in the log-count.
- Results are reported as IRRs and calculated as exp(β), representing the relative change in the occurrence of medication wastage.

For all analyses, estimated coefficients and IRRs were reported with their corresponding 95% confidence intervals (CIs). All statistical analyses were performed using EZR Version 1.70 (Saitama Medical Center, Jichi Medical University, Saitama, Japan) [30]. The level of significance was set at *p* < 0.05.

### Ethical considerations

Ethical approval for this study was not required by the Ethics Committee of Medical Research at the University of Occupational and Environmental Health, Japan, as the research was based on administrative inventory data and did not involve any human subjects or patient-identifiable information.

## Results

**Table 2:** Characteristics of medication wastage in the pre- and post-intervention periods

	Pre-intervention (*n* = 36 months)	Post-intervention (*n* = 48 months)
Total number of medication wastage (cases/month)	301.0 ± 43.0	246.3 ± 26.9
Expired drugs	18.0 ± 7.5 (6.0%)	18.7 ± 5.3 (7.6%)
Change or discontinuation of medication after preparation	112.1 ± 26.1 (37.3%)	82.5 ± 16.9 (33.5%)
Preparation errors or accidental damage	148.4 ± 24.0 (49.3%)	122.2 ± 17.0 (49.6%)
Other	22.5 ± 7.6 (7.5%)	22.9 ± 11.2 (9.3%)
Total cost of medication wastage (JPY/month)	918,759 ± 521,292	552,862 ± 376,174
Expired drugs	352,178 ± 327,205 (38.3%)	255,762 ± 297,111 (46.3%)
Change or discontinuation of medication after preparation	386,029 ± 354,732 (42.0%)	178,470 ± 183,561 (32.3%)
Preparation errors or accidental damage	149,285 ± 179,028 (16.2%)	87,460 ± 74,933 (15.8%)
Other	31,267 ± 33,805 (3.4%)	29,984 ± 51,462 (5.4%)
Number of high-cost medication wastage (cases/month)	3.5 ± 2.0 (1.2%)	2.1 ± 1.7 (0.9%)
Expired drugs	1.5 ± 1.5 (0.5%)	1.0 ± 1.3 (0.4%)
Change or discontinuation of medication after preparation	1.5 ± 1.1 (0.5%)	0.6 ± 0.7 (0.3%)
Preparation errors or accidental damage	0.4 ± 0.5 (0.1%)	0.3 ± 0.5 (0.1%)
Other	0.1 ± 0.4 (0.0%)	0.1 ± 0.4 (0.1%)
Cost of high-cost medication wastage cases (JPY/month)	634,500 ± 505,623 (69.1%)	335,777 ± 362,088 (60.7%)
Expired drugs	263,216 ± 317,914 (28.6%)	182,418 ± 288,165 (33.0%)
Change or discontinuation of medication after preparation	285,510 ± 352,915 (31.0%)	114,941 ± 173,047 (20.8%)
Preparation errors or accidental damage	74,277 ± 175,833 (8.1%)	20,169 ± 59,499 (3.6%)
Other	11,498 ± 29,269 (1.3%)	18,250 ± 49,081 (3.3%)

Abbreviation: JPY, Japanese yen

Data are presented as mean ± standard deviation (SD). Percentages in parentheses represent the proportion of the total medication wastage number or cost.

### Overview of medication wastage characteristics

During the 7-year study period, medication wastage data were consistently recorded. Table 2 summarizes the monthly characteristics during the pre- and post-intervention periods. The average monthly wastage cost decreased from 918,759 JPY to 552,862 JPY. Expired drugs and change or discontinuation of medication after preparation were the primary contributors to the total cost. The number of high-cost medication wastage cases decreased from an average of 3.5 to 2.1 cases per month in the post-intervention period. Supplementary Table 2 presents the detailed annual trends for these data, with the number of high-cost cases further broken down by dosage form. Additionally, the pharmacological subcategories of high-cost medication wastage based on the ATC classification system are shown in Supplementary Table 3, where a decrease in wastage cases was observed for protein kinase inhibitors (L01E) and monoclonal antibodies and antibody-drug conjugates (L01F) following the intervention.

### Primary outcome: impact on total medication wastage costs and estimated cumulative cost savings

**Fig. 1:**
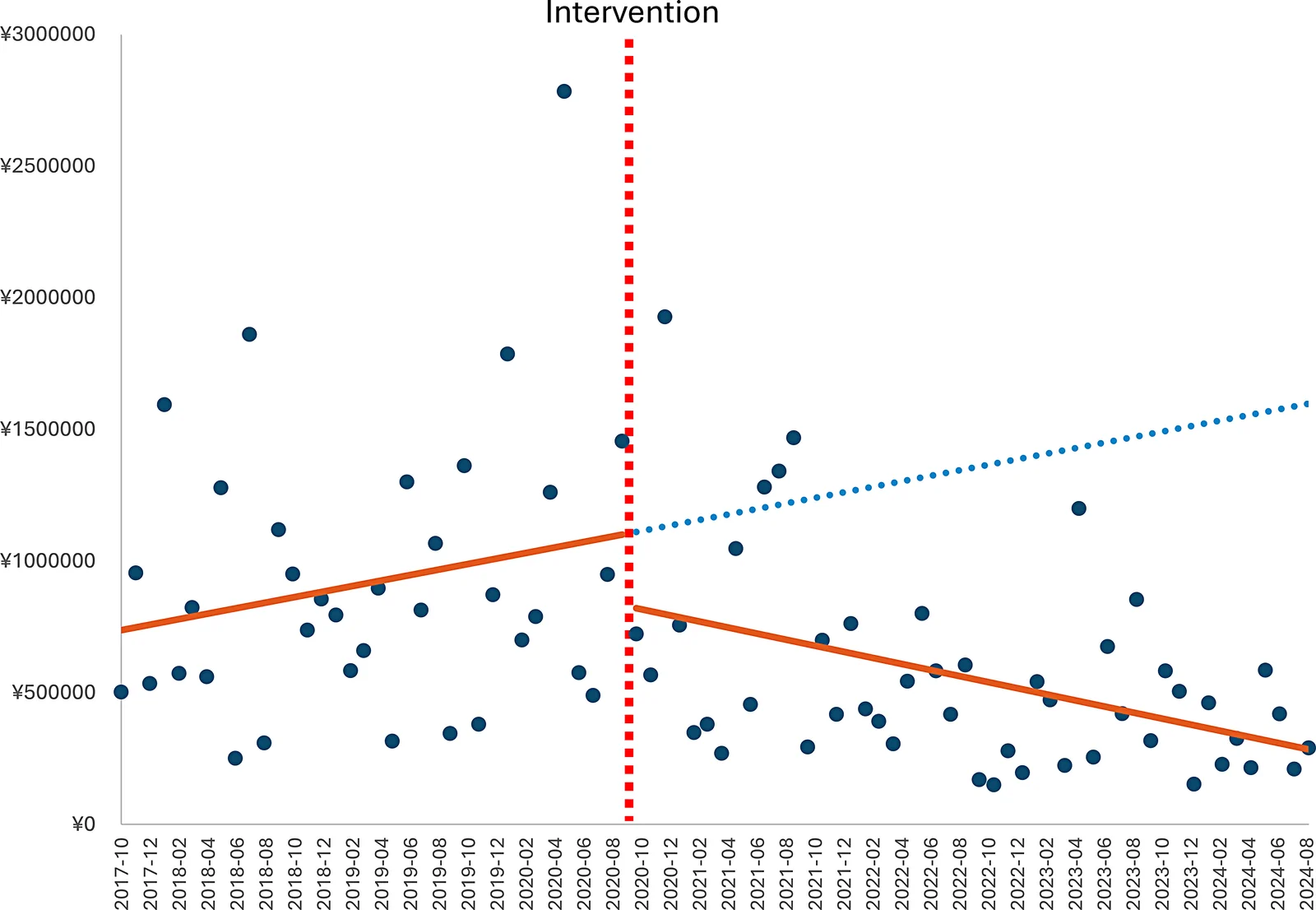
Interrupted time series analysis of total medication wastage costs. The red vertical dashed line indicates the implementation of enhanced pharmacist-led interventions in October 2020. The dots represent the observed monthly total medication wastage costs. The orange solid lines indicate the predicted trends based on the ordinary least squares regression model (linear model), and the blue dashed line represents the counterfactual trend (the projected trend if the intervention had not occurred)

The results of the ITSA for total medication wastage costs are summarized in Fig. 1; Table 3. Prior to the intervention, the baseline monthly wastage cost showed a non-significant increasing trend (β₁ = 10,363 JPY/month; 95% CI, − 3,251 to 23,978; *p* = 0.1338). Following the intervention, there was a non-significant immediate level change (β₂ = −289,669 JPY; 95% CI, − 665,993 to 86,655; *p* = 0.1295). However, a significant change in the post-intervention slope was observed (β₃ = −21,766 JPY/month; 95% CI, − 37,999 to − 5,532; *p* = 0.0092), indicating a decrease compared with the pre-intervention trend. The cumulative cost reduction over the 48-month post-intervention period was estimated to be 38,455,782 JPY.

**Table 3:** Estimated parameters of the interrupted time series analysis of total medication wastage costs

Parameter	Coefficient (Unit)	95% CI	*p*-value
Baseline level (β_0_)	727,036 (JPY)	438,167 to 1,015,904	< 0.0001
Pre-intervention slope (β_1_)	10,363 (JPY/month)	-3,251 to 23,978	0.1338
Level change (β_2_)	-289,669 (JPY)	-665,993 to 86,655	0.1295
Slope change (β_3_)	-21,766 (JPY/month)	-37,999 to -5,532	0.0092

Abbreviations: 95% CI, 95% confidence interval; JPY, Japanese yen

### Secondary outcome: medication wastage costs by reason

**Fig. 2:**
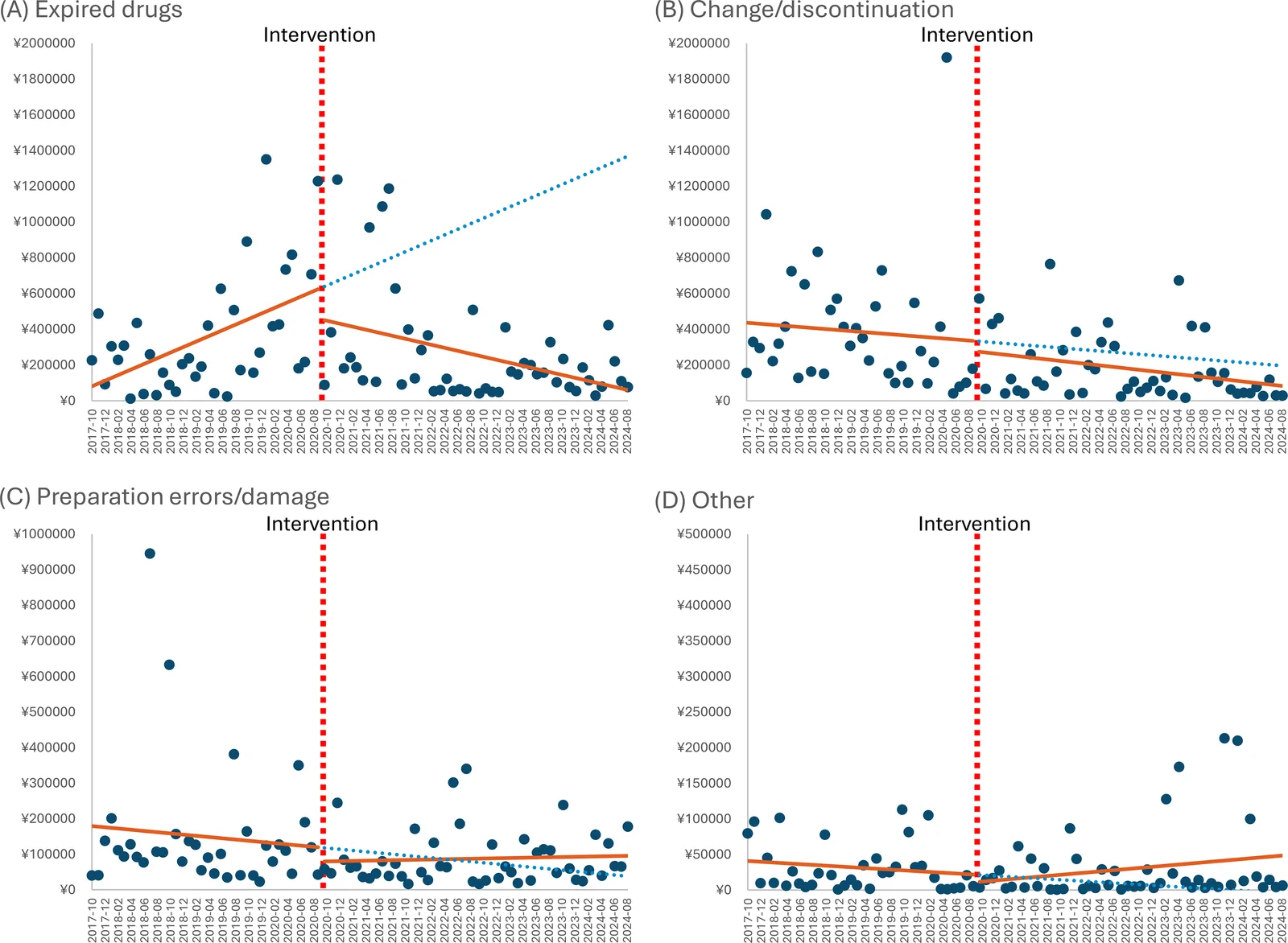
Interrupted time series analysis of medication wastage costs categorized by reason. The four panels display the medication wastage costs categorized by their reason: (**A**) expired drugs; (**B**) change or discontinuation of medication after preparation; (**C**) preparation errors or accidental damage; and (**D**) other. In each panel, the red vertical dashed line indicates the implementation of enhanced pharmacist-led interventions in October 2020. The dots represent the observed monthly medication wastage costs. The orange solid lines indicate the predicted trends based on the ordinary least squares regression model (linear model), and the blue dashed line represents the counterfactual trend (the projected trend if the intervention had not occurred). Note that the scales on the y-axis (costs in Japanese yen) differ among the panels to optimize visualization of trends within each category

The ITSA results categorized by reason are summarized in Fig. 2; Table 4. For expired drugs (Fig. 2A), the pre-intervention trend was a significant monthly increase (β_1_ = 15,508 JPY/month; 95% CI, 6,526 to 24,489; *p* = 0.0009). Post-intervention, there was a significant decrease in the slope (β_3_ = -23,820 JPY/month; 95% CI, -34,530 to -13,111; *p* < 0.0001). For other categories, including change or discontinuation of medication after preparation, preparation errors or accidental damage, and other, no significant changes were observed in the levels or trends (all *p* > 0.1).

**Table 4:** Estimated parameters of the interrupted time series analysis of medication wastage costs by reason

Parameter	Coefficient (Unit)	95% CI	*p*-value
Expired drugs			
Baseline level (β_0_)	65,287 (JPY)	-125,279 to 255,852	0.4973
Pre-intervention slope (β_1_)	15,508 (JPY/month)	6,526 to 24,489	0.0009
Level change (β_2_)	-187,956 (JPY)	-436,216 to 60,303	0.1358
Slope change (β_3_)	-23,820 (JPY/month)	-34,530 to -13,111	< 0.0001
Change or discontinuation of medication after preparation			
Baseline level (β_0_)	439,680 (JPY)	257,240 to 622,119	< 0.0001
Pre-intervention slope (β_1_)	-2,900 (JPY/month)	-11,499 to 5,699	0.5040
Level change (β_2_)	-58,212 (JPY)	-295,886 to 179,461	0.6273
Slope change (β_3_)	-1,172 (JPY/month)	-11,425 to 9,081	0.8206
Preparation errors or accidental damage			
Baseline level (β_0_)	180,739 (JPY)	91,979 to 269,498	0.0001
Pre-intervention slope (β_1_)	-1,700 (JPY/month)	-5,884 to 2,483	0.4210
Level change (β_2_)	-38,288 (JPY)	-153,919 to 77,344	0.5118
Slope change (β_3_)	2,037 (JPY/month)	-2,951 to 7,025	0.4188
Other			
Baseline level (β_0_)	41,331 (JPY)	11,259 to 71,402	0.0077
Pre-intervention slope (β_1_)	-544 (JPY/month)	-1,961 to 873	0.4472
Level change (β_2_)	-9,667 (JPY)	-48,842 to 29,508	0.6247
Slope change (β_3_)	1,329 (JPY/month)	-361 to 3,019	0.1215

Abbreviations: 95% CI, 95% confidence interval; JPY, Japanese yen

### Secondary outcome: impact on high-cost medication wastage

**Fig. 3:**
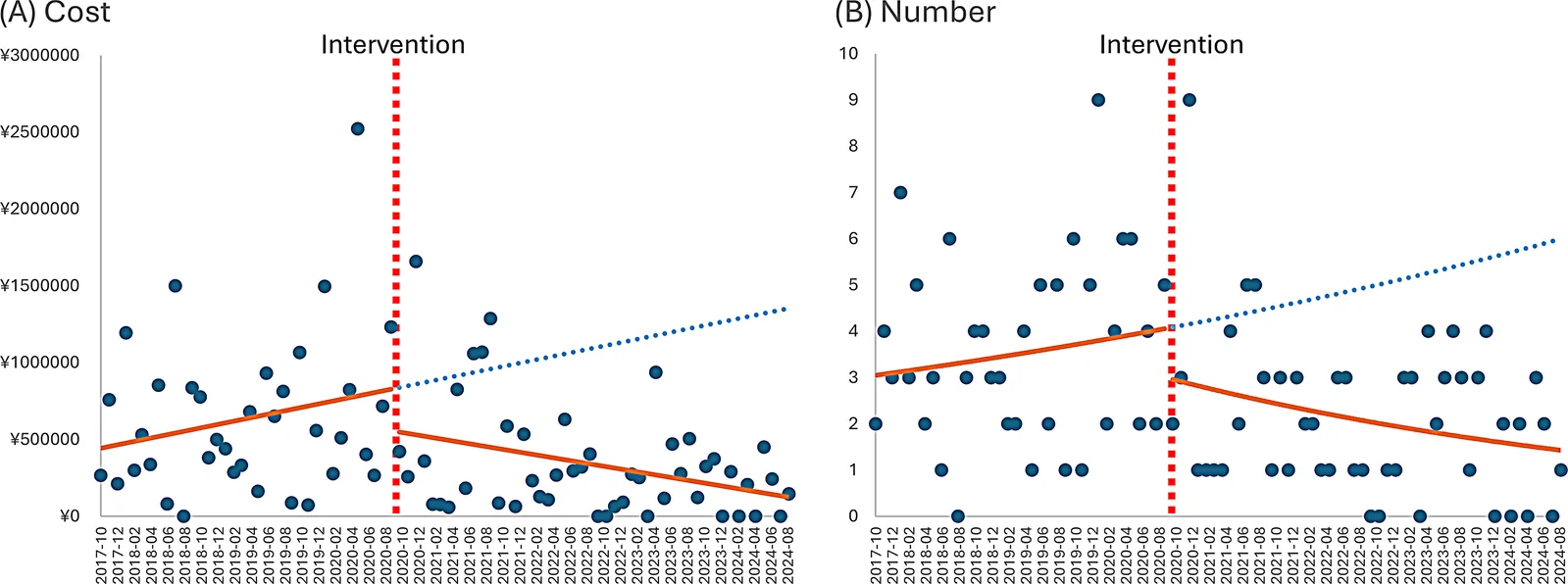
Interrupted time series analysis of high-cost medication wastage. High-cost medication wastage was defined as 50,000 Japanese yen or more per case. The two panels display the high-cost medication wastage in terms of: (**A**) cost and (**B**) number. In each panel, the red vertical dashed line indicates the implementation of enhanced pharmacist-led interventions in October 2020. The dots represent the observed monthly medication wastage costs or numbers. In panel A, the orange solid lines indicate the predicted trends based on the ordinary least squares regression model (linear model), and the blue dashed line represents the counterfactual trend (the projected trend if the intervention had not occurred). In panel B, the orange solid lines indicate the predicted trends based on the Poisson regression with a log-link function (exponential model), and the blue dashed line represents the counterfactual trend (the projected trend if the intervention had not occurred)

The impact on high-cost medication wastage was evaluated in terms of both economic value and frequency (Fig. 3; Tables 5 and 6). In terms of wastage costs, the baseline trend (β_1_ = 10,973 JPY/month; 95% CI, -2,332 to 24,277; *p* = 0.1047) and the immediate level change (β_2_ = -289,567 JPY; 95% CI, -657,313 to 78,180; *p* = 0.1211) were not statistically significant. However, the ITSA revealed a significant post-intervention change in the slope (β_3_ = -20,001 JPY/month; 95% CI, -35,864 to -4,137; *p* = 0.0141). For the number of wastage cases, the Poisson-based ITSA showed a baseline incidence of 3.0 cases per month. Consistent with the cost analysis, the baseline trend (IRR = 1.008; 95% CI, 0.991 to 1.025; p = 0.3412) and immediate level change (IRR = 0.724; 95% CI, 0.442 to 1.184; p = 0.1982) were not significant. A significant decrease in the post-intervention slope was observed (IRR = 0.977; 95% CI, 0.955 to 0.998; *p* = 0.0355). This indicates that the incidence rate of high-cost medication wastage decreased by approximately 2.3% per month following the intervention.

**Table 5:** Estimated parameters of the interrupted time series analysis of high-cost medication wastage costs

Parameter	Coefficient (Unit)	95% CI	*p*-value
Baseline level (β_0_)	431,504 (JPY)	149,220 to 713,789	0.0032
Pre-intervention slope (β_1_)	10,973 (JPY/month)	-2,332 to 24,277	0.1047
Level change (β_2_)	-289,567 (JPY)	-657,313 to 78,180	0.1211
Slope change (β_3_)	-20,001 (JPY/month)	-35,864 to -4,137	0.0141

Abbreviations: 95% CI, 95% confidence interval; JPY, Japanese yen

**Table 6:** Estimated parameters of the interrupted time series analysis of high-cost medication wastage number

Parameter	IRR	95% CI	*p*-value
Baseline level (β_0_)	3.0234	2.0617 to 4.3142	< 0.0001
Pre-intervention slope (β_1_)	1.0082	0.9914 to 1.0253	0.3412
Level change (β_2_)	0.7241	0.4419 to 1.1838	0.1982
Slope change (β_3_)	0.9766	0.9553 to 0.9983	0.0355

Abbreviations: IRR, incidence rate ratio; 95% CI, 95% confidence interval

## Discussion

The aim of this study was to evaluate the long-term economic impact of pharmacist-led initiatives to minimize medication wastage costs using an ITSA. Our results showed that these interventions shifted the trajectory of medication wastage costs from an increasing trend to a sustained decreasing trend, leading to a substantial reduction in wastage costs during the 48-month post-intervention period. To our knowledge, this is the first study to demonstrate the long-term economic efficacy of comprehensive pharmacist-led initiatives on overall hospital-wide medication wastage.

A key finding of this study was that the immediate level change following the intervention was not significant, whereas the change in the long-term post-intervention slope was significant. This result is consistent with the nature of pharmaceutical inventory, as most medications have a shelf life of approximately two to three years at the time of purchase; therefore, the results of improved inventory management and the prevention of new dead stock manifest gradually as older medications eventually reach their expiry dates. Consequently, the significant downward trend observed in the post-intervention period indicates that pharmacist-led measures effectively prevented the accumulation of new waste, leading to sustainable cost reductions.

Our interventions focused specifically on wastage due to expired drugs and high-cost medications, as our previous investigations identified these as primary cost drivers [12, 15]. The pre-intervention period was characterized by an increasing necessity to stock infrequently used medications, a challenge driven by the increase in orphan drugs for rare diseases and the advancement of personalized medicine [31]. Because both types of medications are typically highly expensive, this trend inherently drove up medication costs. To counteract these specific background factors, we implemented targeted initiatives, including the establishment of a “just-in-time” procurement category, the targeted promotion of high-cost, near-expiry drugs, and the mandatory submission of “High-Cost Medication Waste Reports”.

The secondary outcome analysis by wastage reason revealed that our initiatives were particularly effective in reducing costs associated with expired drugs. This finding suggests that our enhanced measures were successful in reducing stockpiles and expired drugs and underscores the principle that effective medication inventory management is essential to minimizing medication wastage. Regarding high-cost medication wastage, although the number of cases accounts for a small proportion of the total, the associated cost represents a large proportion of the total cost. The secondary outcome analysis on high-cost medications showed that reducing the number of wastage cases was directly related to reducing wastage costs, contributing to minimizing total medication wastage costs. According to the ATC classification, high-cost medication wastage cases for protein kinase inhibitors (L01E), which are entirely oral medications and whose wastage was primarily due to expired drugs, fell substantially following the intervention. This reduction can be attributed to the targeted promotion of high-cost, near-expiry drugs, which facilitated their clinical utilization before they expired. Furthermore, for items that eventually became expired drugs, the mandatory submission of “High-Cost Medication Waste Reports” functioned as a prompt to shift those specific agents into the “just-in-time” procurement category for subsequent orders. The combination of these measures successfully minimized the wastage of these high-cost oral medications. A similar reduction was observed under the ATC classification system for monoclonal antibodies and antibody-drug conjugates (L01F) within high-cost medication wastage. These high-cost L01F agents were injectable formulations, and their wastage was predominantly due to the change or discontinuation of medication after preparation. In this context, the mandatory submission of “High-Cost Medication Waste Reports” not only addressed expired drugs but also reduced wastage due to such change or discontinuation of medication after preparation. For example, through this reporting process, in one case where a mixed anticancer drug injection was discontinued because the patient developed a fever, a measure was implemented to check the patient’s body temperature immediately before drug preparation. While it is impossible to completely eliminate medication wastage, especially when caused by unpredictable patient factors, our findings suggest that the mandatory reporting system is an effective tool for heightening awareness among healthcare professionals to prevent the wastage of high-cost medications.

Although the estimated cumulative saving of over 38 million JPY is a model-derived projection based on the counterfactual assumption that the pre-intervention trend would have continued unchanged without the initiatives, rather than a directly observed value, this substantial reduction represents a significant economic benefit from a hospital management perspective. Currently, hospital operating costs are rising due to factors such as inflation, increased labor costs, and escalating drug prices. Our findings underscore the pivotal role pharmacists can play in minimizing unnecessary expenditures.

Additional strategies to further minimize medication wastage could include strengthening pharmacist-led reviews of the patient’s condition, such as assessing their condition using laboratory data, prior to the preparation of expensive anticancer drugs [21, 22]. Furthermore, proactive education of other healthcare professionals by pharmacists regarding wastage minimization could yield further benefits [32, 33].

This study has several limitations. First, this was a single-center study conducted at a university hospital offering advanced treatments; therefore, the generalizability of our results to other healthcare settings may be limited. Second, we implemented various interventions; however, the specific impact of each individual measure could not be isolated. Third, although no additional pharmacy staff were hired, the operational costs associated with implementing and maintaining these initiatives were not quantified. Specifically, the evaluation did not account for the increased workload and time required for pharmacists to conduct the targeted promotion of near-expiry drugs, or for healthcare professionals to prepare the mandatory “High-Cost Medication Waste Reports”. However, given the scale of cost savings, the economic benefits likely far outweigh these unmeasured operational costs. Fourth, while the structure of ward-based pharmacy services remained unchanged during the study period, clinical pharmacists contributed to the initiatives, such as by sharing information on scheduled non-stock medication use to facilitate the “just-in-time” procurement category. However, the quantitative impact of these ward-based services could not be evaluated in this study. Finally, the study period (2017–2024) overlapped with the COVID-19 pandemic, which may have influenced hospital activity and medication usage patterns. To account for these effects, we initially included the monthly medication purchase amount and the inpatient days as covariates for hospital activity in the ITSA model; however, the stepwise selection process based on the AIC indicated that excluding this variable resulted in a better model fit. This suggests that the observed reduction in medication wastage costs was primarily driven by our pharmacist-led initiatives rather than pandemic-related changes in hospital volume. Nevertheless, despite the robustness of the ITSA method, we cannot entirely rule out the influence of this and other unmeasured factors during the 7-year period.

## Conclusion

This study demonstrated that pharmacist-led initiatives focusing on expired and high-cost medications achieved a significant and sustained long-term reduction in medication wastage costs. These findings underscore the pivotal role of pharmacists in enhancing hospital financial sustainability by effectively mitigating avoidable medication-related economic losses. 

## Supplementary Information

Below is the link to the electronic supplementary material.

Supplementary Material 1

Supplementary Material 2

Supplementary Material 3

## Data Availability

The datasets generated and analyzed during the current study are not publicly available due to institutional restrictions regarding financial and pharmaceutical data but are available from the corresponding author on reasonable request.

## Abbreviations

AIC: Akaike Information Criterion
ATC: Anatomical Therapeutic Chemical
IRR: Incidence rate ratio
ITSA: Interrupted time series analysis
JPY: Japanese yen
VIF: Variance inflation factor
95% CI: 95% confidence interval
