# Supplementary material for: Economic impact of pharmacist-led initiatives to minimize medication wastage costs in the hospital setting: an interrupted time series analysis: J. Pharm. Health Care Sci. (Research article)
Source: J Pharm Health Care Sci. 2026 Jul 28;12:92. doi: 10.1186/s40780-026-00612-z (PMC13700122; doi:10.1186/s40780-026-00612-z)
Supplement: Supplementary file 1 [file 40780_2026_612_MOESM1_ESM.docx]

| Supplementary Table 1 Sensitivity analysis using alternative interrupted time series analysis models | | | | |
| --- | --- | --- | --- | --- |
| (a) Model incorporating both the monthly purchase amount and the monthly inpatient days | | | | |
| Parameter | Coefficient (Unit) | 95% CI | *p*-value | VIF |
| Baseline level (β_0_) | 585459 (JPY) | -786278 to 1957197 | 0.3981 | — |
| Pre-intervention slope (β_1_) | 12536 (JPY/month) | -5209 to 30280 | 0.1636 | 21.0765 |
| Level change (β_2_) | -313081 (JPY) | -712871 to 86709 | 0.1230 | 4.4567 |
| Slope change (β_3_) | -23173 (JPY/month) | -43806 to -2540 | 0.0282 | 11.8709 |
| Purchase amount (β_4_) | -0.0003 (—) | -0.0027 to 0.0022 | 0.8136 | 6.6424 |
| Inpatient days (β_5_) | 13.3989 (JPY/inpatient day) | -57.5720 to 84.3698 | 0.7080 | 1.8515 |
| (b) Model incorporating the monthly purchase amount alone | | | | |
| Parameter | Coefficient (Unit) | 95% CI | *p*-value | VIF |
| Baseline level (β_0_) | 770570 (JPY) | -183338 to 1724478 | 0.1118 | — |
| Pre-intervention slope (β_1_) | 10526 (JPY/month) | -3592 to 24645 | 0.1418 | 13.4949 |
| Level change (β_2_) | -290285 (JPY) | -669254 to 88683 | 0.1313 | 4.0502 |
| Slope change (β_3_) | -21380 (JPY/month) | -39594 to -3166 | 0.0220 | 9.3562 |
| Purchase amount (β_4_) | -0.0001 (—) | -0.0023 to 0.0021 | 0.9243 | 5.5902 |
| Abbreviations: 95% CI, 95% confidence interval; JPY, Japanese yen; VIF, variance inflation factor | | | | |
