# Supplementary material for: Economic impact of pharmacist-led initiatives to minimize medication wastage costs in the hospital setting: an interrupted time series analysis: J. Pharm. Health Care Sci. (Research article)
Source: J Pharm Health Care Sci. 2026 Jul 28;12:92. doi: 10.1186/s40780-026-00612-z (PMC13700122; doi:10.1186/s40780-026-00612-z)
Supplement: Supplementary file 2 [file 40780_2026_612_MOESM2_ESM.docx]

| Supplementary Table 2 Annual summary of medication wastage | | | | | | | |
| --- | --- | --- | --- | --- | --- | --- | --- |
|  | Pre-intervention | | | Post-intervention | | | |
|  | Oct-2017 to Sep-2018 | Oct-2018 to Sep-2019 | Oct-2019 to Sep-2020 | Oct-2020 to Sep-2021 | Oct-2021 to Sep-2022 | Oct-2022 to Sep-2023 | Oct-2023 to Sep-2024 |
| Total number of medication wastage (cases/year) | 3859 | 3764 | 3214 | 3112 | 2931 | 2763 | 3018 |
| - Expired drugs | 171 | 195 | 281 | 263 | 213 | 212 | 210 |
| - Change or discontinuation | 1515 | 1391 | 1131 | 1140 | 1035 | 894 | 890 |
| - Preparation errors or damage | 1891 | 1891 | 1562 | 1502 | 1478 | 1386 | 1500 |
| - Others | 282 | 287 | 240 | 207 | 205 | 271 | 418 |
| Total cost of medication wastage (JPY/year) | 10357633 | 9317673 | 13400030 | 10504745 | 6254372 | 5431559 | 4289743 |
| - Expired drugs | 2580651 | 2701050 | 7396701 | 6415228 | 2180579 | 1974107 | 1706647 |
| - Change or discontinuation | 5280601 | 4446439 | 4170018 | 3008532 | 2449309 | 2216989 | 891729 |
| - Preparation errors or damage | 2078336 | 1880477 | 1415457 | 865982 | 1412801 | 832344 | 1086946 |
| - Others | 418045 | 289707 | 417854 | 215004 | 211684 | 408118 | 604421 |
| Number of high-cost cases (cases/year) | 39  (31,7,1) | 36  (28,8,-) | 52  (39,13,-) | 37  (21,16,-) | 22  (17,5,-) | 24  (19,5,-) | 18  (14,3,1) |
| - Expired drugs | 12  (4,7,1) | 11  (3,8,-) | 31  (18,13,-) | 25  (9,16,-) | 7  (2,5,-) | 12  (7,5,-) | 6  (3,2,1) |
| - Change or discontinuation | 22  (22,-,-) | 20  (20,-,-) | 13  (13,-,-) | 9  (9,-,-) | 10  (10,-,-) | 7  (7,-,-) | 4  (3,1,-) |
| - Preparation errors or damage | 4  (4,-,-) | 4  (4,-,-) | 5  (5,-,-) | 2  (2,-,-) | 4  (4,-,-) | 3  (3,-,-) | 5  (5,-,-) |
| - Others | 1  (1,-,-) | 1  (1,-,-) | 3  (3,-,-) | 1  (1,-,-) | 1  (1,-,-) | 2  (2,-,-) | 3  (3,-,-) |
| Cost of high-cost cases (JPY/year) | 6862720 | 6035232 | 9944061 | 7329415 | 3653958 | 2977587 | 2156344 |
| - Expired drugs | 1881580 | 1677620 | 5916561 | 5076192 | 1651892 | 996234 | 1031756 |
| - Change or discontinuation | 3881620 | 3262974 | 3133770 | 2006952 | 1618913 | 1560997 | 330285 |
| - Preparation errors or damage | 1004910 | 1020988 | 648080 | 187499 | 320604 | 134353 | 325643 |
| - Others | 94610 | 73650 | 245650 | 58772 | 62549 | 286003 | 468660 |
| Abbreviation: JPY, Japanese yen  The numbers in parentheses indicate, in order, the counts of injections, oral preparations, and topical preparations, classified by dosage form. | | | | | | | |
