# Supplementary material for: Economic impact of pharmacist-led initiatives to minimize medication wastage costs in the hospital setting: an interrupted time series analysis: J. Pharm. Health Care Sci. (Research article)
Source: J Pharm Health Care Sci. 2026 Jul 28;12:92. doi: 10.1186/s40780-026-00612-z (PMC13700122; doi:10.1186/s40780-026-00612-z)
Supplement: Supplementary file 3 [file 40780_2026_612_MOESM3_ESM.docx]

| Supplementary Table 3 Drug types for medication wastage in high-priced medications | | | | | | | | | |
| --- | --- | --- | --- | --- | --- | --- | --- | --- | --- |
| Drug types | | | Pre-intervention | | | Post-intervention | | | |
| First level (Anatomical main group) | Second level (Therapeutic subgroup) | Third level (Pharmacological subgroup) | Oct-2017 to Sep-2018 | Oct-2018 to Sep-2019 | Oct-2019 to Sep-2020 | Oct-2020 to Sep-2021 | Oct-2021 to Sep-2022 | Oct-2022 to Sep-2023 | Oct-2023 to Sep-2024 |
| A:  Alimentary tract and metabolism | A07:  Antidiarrheals, intestinal antiinflammatory/antiinfective agents | A07A:  Intestinal antiinfectives |  |  |  |  | 1  (1,-,-,-) |  |  |
|  | A16:  Other alimentary tract and metabolism products | A16A:  Other alimentary tract and metabolism products |  |  | 1  (1,-,-,-) | 1  (1,-,-,-) |  |  | 1  (-,1,-,-) |
| B:  Blood and blood-forming organs | B01:  Antithrombotic agents | B01A:  Antithrombotic agents | 4  (1,1,2,-) | 5  (1,2,2,-) | 7  (3,3,1,-) | 2  (1,1,-,-) | 3  (1,1,1,-) |  | 2  (2,-,-,-) |
|  | B02:  Antihemorrhagics | B02B:  Vitamin K and other hemostatics |  | 1  (-,1,-,-) | 3  (2,-,1,-) | 2  (2,-,-,-) | 1  (-,1,-,-) | 1  (-,1,-,-) | 1  (1,-,-,-) |
|  | B06:  Other hematological agents | B06A:  Other hematological agents |  |  | 1  (1,-,-,-) |  |  |  |  |
| C:  Cardiovascular system | C01:  Cardiac therapy | C01E:  Other cardiac preparations |  |  |  | 1  (1,-,-,-) |  |  |  |
|  | C04:  Peripheral vasodilators | C04A:  Peripheral vasodilators |  |  |  |  |  | 1  (-,-,1,-) | 1  (-,-,1,-) |
| D:  Dermatologicals | D11:  Other dermatological preparations | D11A:  Other dermatological preparations |  |  | 2  (-,-,1,1) | 1  (-,-,-,1) |  |  |  |
| H:  Systemic hormonal preparations, excl. sex hormones and insulins | H01:  Pituitary and hypothalamic hormones and analogues | H01A:  Anterior pituitary lobe hormones and analogues |  |  | 2  (2,-,-,-) |  |  |  |  |
|  |  | H01C:  Hypothalamic hormones |  |  |  |  |  | 1  (1,-,-,-) |  |
| J:  Antiinfectives for systemic use | J01:  Antibacterials for systemic use | J01X:  Other antibacterials |  | 1  (1,-,-,-) |  |  |  |  |  |
|  | J02:  Antimycotics for systemic use | J02A:  Antimycotics for systemic use | 1  (1,-,-,-) | 1  (1,-,-,-) |  |  |  | 1  (1,-,-,-) | 2  (1,-,1,-) |
|  | J04:  Antimycobacterials | J04A:  Drugs for treatment of tuberculosis |  |  |  | 1  (1,-,-,-) |  |  |  |
|  | J05:  Antivirals for systemic use | J05A:  Direct acting antivirals | 2  (2,-,-,-) | 3  (3,-,-,-) | 5  (5,-,-,-) | 1  (1,-,-,-) |  | 2  (1,-,1,-) |  |
|  | J06:  Immune sera and immunoglobulins | J06B:  Immunoglobulins | 2  (-,2,-,-) |  | 1  (-,1,-,-) | 1  (-,-,1,-) |  |  | 1  (-,1,-,-) |
|  | J07:  Vaccines | J07B:  Viral vaccines |  |  | 1  (1,-,-,-) | 1  (1,-,-,-) |  |  |  |
| L:  Antineoplastic and immunomodulating agents | L01:  Antineoplastic agents | L01A:  Alkylating agents | 2  (2,-,-,-) |  | 3  (2,-,1,-) | 1  (1,-,-,-) |  | 1  (-,1,-,-) |  |
|  |  | L01B:  Antimetabolites | 2  (-,2,-,-) |  | 1  (-,1,-,-) |  | 1  (-,-,1,-) |  | 1  (-,-,1,-) |
|  |  | L01C:  Plant alkaloids and other natural products | 1  (-,1,-,-) | 3  (-,3,-,-) |  | 1  (-,1,-,-) |  |  |  |
|  |  | L01E:  Protein kinase inhibitors | 1  (1,-,-,-) | 2  (2,-,-,-) | 8  (8,-,-,-) | 11  (11,-,-,-) | 1  (1,-,-,-) | 2  (2,-,-,-) | 1  (1,-,-,-) |
|  |  | L01F:  Monoclonal antibodies and antibody drug conjugates | 15  (-,14,1,-) | 13  (-,11,2,-) | 6  (-,6,-,-) | 4  (-,4,-,-) | 9  (-,7,2,-) | 5  (-,5,-,-) | 2  (-,1,-,1) |
|  |  | L01X:  Other antineoplastic agents | 2  (1,1,-,-) | 3  (1,1,-,1) | 3  (1,1,1,-) | 4  (3,1,-,-) |  | 2  (2,-,-,-) | 1  (1,-,-,-) |
|  | L02:  Endocrine therapy | L02A:  Hormones and related agents | 1  (-,-,1,-) |  |  |  |  |  |  |
|  |  | L02B:  Hormone antagonists and related agents | 1  (1,-,-,-) |  |  |  |  |  |  |
|  | L03:  Immunostimulants | L03A:  Immunostimulants | 3  (2,-,-,1) |  | 1  (-,-,-,1) | 1  (1,-,-,-) |  | 1  (1,-,-,-) | 1  (-,-,-,1) |
|  | L04:  Immunosuppressants | L04A:  Immunosuppressants | 1  (-,1,-,-) | 1  (1,-,-,-) | 5  (4,-,-,1) | 2  (-,2,-,-) | 4  (2,1,-,1) | 3  (-,-,1,2) | 1  (-,-,-,1) |
| N:  Nervous system | N02:  Analgesics | N02A:  Opioids | 1  (1,-,-,-) |  |  |  |  | 1  (1,-,-,-) |  |
|  | N03:  Antiepileptics | N03A:  Antiepileptics |  |  |  |  | 1  (1,-,-,-) |  |  |
|  | N07:  Other nervous system drugs | N07X:  Other nervous system drugs |  |  |  |  |  | 1  (1,-,-,-) | 1  (-,1,-,-) |
| S:  Sensory organs | S01:  Ophthalmologicals | S01L:  Ocular vascular disorder agents |  | 2  (-,2,-,-) | 1  (-,1,-,-) | 1  (-,-,1,-) |  |  | 2  (-,-,2,-) |
| V:  Various | V03:  All other therapeutic products | V03A:  All other therapeutic products |  | 1  (1,-,-,-) | 1  (1,-,-,-) | 1  (1,-,-,-) | 1  (1,-,-,-) | 2  (2,-,-,-) |  |
| Values represent the number of medication wastage cases of 50,000 JPY or more per case. Drug types were classified according to the Anatomical Therapeutic Chemical (ATC) classification system, with the analysis covering ATC codes down to the third level (pharmacological subgroup). Monteplase, thrombomodulin, and antithrombin gamma, which are not assigned ATC codes, are classified as B01A based on their pharmacological actions in this study. The numbers in parentheses indicate, in order, the counts for each reason: expired drugs, change or discontinuation, preparation errors or damage, and other. | | | | | | | | | |
